# Proteomic Analysis of Myocardia Containing the Obscurin R4344Q Mutation Linked to Hypertrophic Cardiomyopathy

**DOI:** 10.3389/fphys.2020.00478

**Published:** 2020-05-18

**Authors:** Li-Yen R. Hu, Aikaterini Kontrogianni-Konstantopoulos

**Affiliations:** Department of Biochemistry and Molecular Biology, University of Maryland School of Medicine, Baltimore, MD, United States

**Keywords:** obscurin, cardiomyopathy, tandem mass-tagged mass spectrometry, phosphoproteomics, cytoskeleton, Ca^2+^ cycling, metabolism, ion and molecular transport

## Abstract

Obscurin is a giant cytoskeletal protein with structural and regulatory roles encoded by the *OBSCN* gene. Recently, mutations in *OBSCN* were associated with the development of different forms of cardiomyopathies, including hypertrophic cardiomyopathy (HCM). We previously reported that homozygous mice carrying the HCM-linked R4344Q obscurin mutation develop arrhythmia by 1-year of age under sedentary conditions characterized by increased heart rate, frequent incidents of premature ventricular contractions, and episodes of spontaneous ventricular tachycardia. In an effort to delineate the molecular mechanisms that contribute to the observed arrhythmic phenotype, we subjected protein lysates prepared from left ventricles of 1-year old R4344Q and wild-type mice to comparative proteomics analysis using tandem mass spectrometry; raw data are available via ProteomeXchange with identifier PXD017314. We found that the expression levels of proteins involved in cardiac function and disease, cytoskeletal organization, electropotential regulation, molecular transport and metabolism were significantly altered. Moreover, phospho-proteomic evaluation revealed changes in the phosphorylation profile of Ca^2+^ cycling proteins, including sAnk1.5, a major binding partner of obscurin localized in the sarcoplasmic reticulum; notably, this is the first report indicating that sAnk1 undergoes phosphorylation. Taken together, our findings implicate obscurin in diverse cellular processes within the myocardium, which is consistent with its multiple binding partners, localization in different subcellular compartments, and disease association.

## Introduction

Muscle contraction is a highly regulated process that depends on the coordinated assembly of the sarcomeric cytoskeleton and internal membrane systems regulating Ca^2+^ cycling. As such, giant scaffolding proteins guide the incorporation of myofilament and accessory proteins into striated structures and contribute to the sarcomeric alignment of the sarcoplasmic reticulum (SR) and transverse tubules (t-tubules) to form functional couplons where excitation-contraction (EC) takes place (Wang et al., 2018). Given that EC is an energy demanding process, enzymes supplying adequate levels of metabolites are also targeted to the mature sarcomere in order to sustain repetitive contractions, and thus the ability of the heart to beat incessantly (Hu et al., 2015).

Obscurin, encoded by the *OBSCN* gene, is a giant scaffolding protein surrounding sarcomeric Z-disks and M-bands. It is highly modular consisting of 68 immunoglobulin (Ig) and 3 fibronectin-III (Fn-III) domains in addition to an array of signaling motifs that include an isoleucine glutamine (IQ) motif that binds calmodulin, a Src homology 3 (SH3) domain, a Rho-guanine nucleotide exchange factor (Rho-GEF) domain in tandem with a pleckstrin homology (PH) domain, and two active Ser/Thr kinase domains (Wang et al., 2018). Given its unique position at the periphery of Z-disks and M-bands, obscurin mediates binding to structural, regulatory and membrane-associated proteins residing in different subcellular compartments via its adhesion and signaling motifs (Bagnato et al., 2003; Kontrogianni-Konstantopoulos et al., 2003; Bowman et al., 2007; Fukuzawa et al., 2008; Ackermann et al., 2009; Ford-Speelman et al., 2009; Hu and Kontrogianni-Konstantopoulos, 2013; Randazzo et al., 2013; Qadota et al., 2016; Shriver et al., 2016). Through these binding interactions, obscurin plays key roles in the proper incorporation of myosin thick filaments into A-bands and the stability and maintenance of A- and M-bands (Kontrogianni-Konstantopoulos et al., 2006; Ackermann et al., 2009), the subsarcolemmal organization of the microtubule network and dystrophin into costameres (Randazzo et al., 2013), cell adhesion (Hu and Kontrogianni-Konstantopoulos, 2013), stress/stretch and growth responses (Ackermann et al., 2017; Randazzo et al., 2017), the organization and cytoskeletal anchoring of the SR membranes (Bagnato et al., 2003; Kontrogianni-Konstantopoulos et al., 2003; Lange et al., 2009), and Ca^2+^ homeostasis (Spooner et al., 2012; Hu et al., 2017).

Recent studies have further highlighted the essential roles of obscurin in cardiac pathophysiology as missense and frameshift mutations in the *OBSCN* gene were shown to co-segregate with hypertrophic cardiomyopathy (HCM), dilated cardiomyopathy (DCM) and left ventricular non-compaction (LVNC) (Grogan and Kontrogianni-Konstantopoulos, 2018). Although the etiologies that underlie the pathogenicity of the majority of these mutations are elusive, work from our group has demonstrated that homozygous knock-in female mice carrying the HCM-linked R4344Q mutation located in Ig58 develop ventricular arrhythmia by 1-year of age under sedentary conditions, compensatory HCM in the presence of mild operational stress, and a DCM-like phenotype following exertion of sustained mechanical stress (Hu et al., 2017). Our biochemical and electrophysiological studies further showed that Ca^2+^ deregulation underlies at least in part these phenotypic manifestations as mutant obscurin (containing the R4344Q substitution) exhibits enhanced binding to phospholamban (PLN), the major regulator of the sarco/endoplasmic reticulum Ca^2+^ adenosine triphosphatase 2 (SERCA2), resulting in increased SR Ca^2+^ load and contractility kinetics, likely due to sequestration of PLN and disinhibition of SERCA2 (Hu et al., 2017).

Given the cardiac pathology that the R4344Q knock-in animals develop, we sought insights into the molecular pathways and cellular processes that are altered in the mutant myocardium. To this end, we performed an in-depth proteomics analysis coupled with evaluation of post-translational alterations using protein lysates prepared from left ventricles of sedentary, 1-year old female knock-in and wild-type animals. Our studies reveal alterations in the expression levels and post-translational regulation via phosphorylation of proteins involved in major pathophysiological processes and highlight the involvement of obscurin in their modulation.

## Materials and Methods

All animal care, experimental procedures, and methodologies were performed in accordance with the protocols approved by the Institutional Animal Care and Use Committees of the University of Maryland School of Medicine.

### Sample Preparation

Isogenic 1-year old female wild-type (*n* = 3) and obscurin*^R4344Q^* knock-in (*n* = 3) mice (Hu et al., 2017) were perfused with phosphate buffered saline (PBS) supplemented with a complete Mini Protease Inhibitor Cocktail (Roche, Indianapolis, IN, United States). The hearts were excised, and flash-frozen in liquid nitrogen. The left ventricles were isolated and used for generation of protein lysates with freshly prepared urea-thiourea buffer containing 8M urea, 2M thiourea, 0.05M Tris-HCl, pH 6.8, 75mM dithiothreitol, 3% (w/v) sodium dodecyl sulfate, 10% (v/v) glycerol supplemented with Halt Protease Inhibitor Cocktail (Thermo Fisher Scientific, Waltham, MA, United States) and Halt Phosphatase Inhibitor Cocktail (Thermo Fisher Scientific). Hundred μg of protein lysates from each left ventricle were reduced with 4.5 mM tris-(2-carboxyethyl) phosphine (TCEP) (Thermo Fisher Scientific) and subjected to alkylation with 4.3 mM methyl methanethiosuphonate (Thermo Fisher Scientific). The alkylated lysates were precipitated with 10% (v/v) trichloroacetic acid in acetone (Sigma-Aldrich, St. Louis, MO, United States). The precipitated protein samples were reconstituted in 100 μl of 100 mM tri-ethyl-ammonium bicarbonate (TEAB), pH 8, and digested with 4 μg of trypsin/LysC mixture (V5071, Promega, Madison, WI, United States) at 37°C overnight. Peptides were labeled with 0.8 mg Tandem Mass Tag (TMT) 10-plex Isobaric Label Reagent (Thermo Fisher Scientific) in 100 mM TEAB, according to the manufacturer’s instructions. The isobaric nature of TMT allows the same peptides collected from different samples to co-migrate in liquid chromatography but be resolved by mass spectrometry. Thus, this approach allows quantification of the relative abundance of peptides from multiple samples, and enhances the signal-to-noise ratio for the identification of a particular peptide (Thompson et al., 2003).

### Basic Reverse Phase Fractionation

One third of combined TMT 10-plex labeled tryptic peptides from each sample (i.e., wild-type and obscurin*^R4344Q^* knock-in) was reconstituted in 2 ml of buffer A (10 mM TEAB in H_2_0, pH 8) and subjected to basic Reverse Phase (bRP) fractionation (Johns Hopkins University Mass Spectrometry and Proteomic Facility). The injection rate of the samples was set at 250 μl/min over 8 min. Peptides were fractionated on a 5 μm/2.1 × 100 mm XBridge C18 Column (Waters, Milford, MA, United States) using a high-performance liquid chromatography (HPLC) system (Agilent, Santa Clara, CA, United States). Fractionation was carried out by a linear gradient between buffer A and buffer B (10 mM TEAB in 90% acetonitrile) with the flow rate set at 250 μl/min. Eighty-four (84) fractions of 225 μl were collected between 20 and 95 min with 0–100% of buffer B. The 84 fractions were re-combined in 24 fractions that were analyzed by liquid chromatography/tandem mass spectrometry.

### Liquid Chromatography/Tandem Mass Spectrometry

Each of the 24 bRP fractions was reconstituted in 28 μl of 2% acetonitrile/0.1% formic acid. Five hundred nanograms (500 ng) per fraction were subjected to reverse phase chromatography in a 2–90% acetonitrile/0.1% formic acid gradient over 90 min at 300 nl/min on a 5 μm/120 Å/75 μm × 150 mm ProntoSIL-120-5-C18 H column (Bischoff Chromatography, Munich, Germany), and subsequently sprayed on a nano-LC-Q Exactive HF (Thermo Fisher Scientific) through 1 μm emitter tip (Scientific Instrument Services Inc, Ringoes, NJ, United States) at 2.0 kV interfaced with the nanoAcquity LC system (Waters). Survey scans (including full mass spectra) were acquired on Orbi-trap within 350–1700 Da m/z using the Data dependent Top 15 method with dynamic exclusion of 20 s. Precursor ions were individually isolated with 1.0 Da and fragmented using Higher energy Collision activated Dissociation (HCD) activation collision energy of 32. Precursor and fragment ions were analyzed at 120,000 resolution, 3xe^6^ Automatic Gain Control (AGC) target and 50 ms maximum Injection Time (max IT), and at 60,000 resolution, 1xe^5^ AGC target and 200 ms max IT, respectively.

### Data Analysis

MS/MS spectra were analyzed with the Proteome Discoverer (PD) software (v1.4 Thermo Fisher Scientific) using 3Nodes; common PD nodes with spectra were extracted, charge state deconvoluted, and deisotoped using Xtract option at resolution 100K at 400Da in an MS2 processor. MS/MS spectra from 3Nodes were analyzed with Mascot (v2.5.1, Matrix Science, London, United Kingdom) with the RefSeq2015 Complete Database (*Mus musculus* species, trypsin as an enzyme, mc2). We performed two searches for post-translational modifications. Both searches accounted for TMT10plex on the NH_2_-terminus and carbamidomethylation on cysteine as static modifications, and TMT on lysine, oxidation on methionine and deamidation on QN as variable modifications. In addition, the first search included phosphorylation on serine and threonine, and the phosphorylation sites were identified with both Mascot and PhosphoRS4.1. The second search included *O*-GlcNAcylation on serine and threonine, and glutathionylation on cysteine. Peptides identified from Mascot searches were processed with PD to match to corresponding proteins with 0.01% threshold of False Discover Rate (FDR) based on a concatenated decoy database search to calculate protein and peptide ratios. The mass spectra reporter ion intensities ascertained from PD provided multiple spectra values for all the detected modified peptides of all samples. The median signal value of all intensities for each peptide’s modification class, e.g., phosphorylated or dephosphorylated, was calculated and converted to log2 notation (with 0.0 values excluded as nulls) to represent that peptide and its modification state for subsequent analysis. To minimize any potential experimental artifact effect due to differential sample processing and/or loading, these peptide values were quantile-normalized across samples so that all samples’ modified peptide values had the same median and distributions. A two-tailed *t*-test using a one-way ANOVA model was applied to evaluate the adjusted modified peptides between wild-type and R4344Q LV samples using the Partek Genomics Suite 6.6 analytic platform (Partek Inc. St. Louis, MO, United States). The 95% confidence interval of the linear fold-change provided by the Partek Genomics Suite analytic platform was converted to relative ratio change. Functions and pathways of proteins with altered expression levels were analyzed with Ingenuity Pathway Analysis (IPA) (Qiagen, Hilden, Germany).

### Data Repository

The raw mass spectrometry proteomics data have been deposited to the ProteomeXchange Consortium via the PRIDE partner repository (Perez-Riverol et al., 2019) with the dataset identifier PXD017314. In addition, all proteins identified from the mass spec analysis along with the relative statistics (i.e., knock-in *vs.* wild-type) regardless of whether they were deemed significantly altered or not are included in Supplementary File S1.

## Results and Discussion

The R4344Q obscurin mutation was originally identified via genetic screening in a young adult Japanese patient presenting with HCM (Arimura et al., 2007). The patient’s mother was also diagnosed with HCM, and although she was not screened for the mutation, it was postulated that the index patient most likely inherited the mutation from her since the father did not carry it and did not develop HCM (Arimura et al., 2007).

To study the disease pathogenesis due to the R4344Q mutation, we recently generated a knock-in mouse model carrying the mutation (Hu et al., 2017). Examination of female homozygous knock-in mice indicated that they exhibit increased heart rate, frequent incidents of premature ventricular contractions, and episodes of spontaneous ventricular tachycardia at 1-year of age under sedentary conditions (Hu et al., 2017); of note, heterozygous knock-in mice did not show any cardiac pathology. Exertion of sustained mechanical stress induced via transaortic constriction (TAC) surgery in young R4344Q knock-in mice resulted in the development of a DCM-like phenotype, which was associated with increased fibrosis and calcification (Hu et al., 2017). Interestingly, sham knock-in animals subjected to surgery but not TAC developed HCM, possibly as an adaptive response (Hu et al., 2017). Taken together, these observations indicated that the presence of the R4344Q mutation results in cardiac remodeling and dysfunction in response to the physiological process of aging and following mild or sustained pathological stress in the young.

In the current study, we focused on the molecular alterations taking place in the R4344Q myocardia as a function of aging under sedentary conditions. To identify differences in the relative abundance and post-translational regulation of key proteins contributing to the observed arrhythmic phenotype, we performed an unbiased, in-depth, comparative proteomics analysis using left ventricles (LV) from 1-year old isogenic wild-type (*n* = 3) and R4344Q knock-in (*n* = 3) homozygous female mice. We identified 4,343 proteins via our proteomic screening, 181 of which exhibited significantly altered expression (Supplementary File S1). In addition to changes in the expression levels of these 181 proteins, we found alterations in the post-translational regulation of select ones, with phosphorylation being the major modification.

**FIGURE 1:**
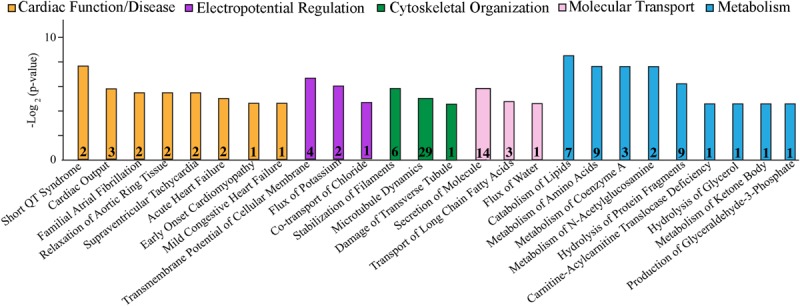
Cellular process/disease annotation of proteins with altered expression in left ventricles of 1-year-old R4344Q knock-in female mice. The expression levels of proteins involved in cardiac function and disease (orange), electropotential regulation (purple), cytoskeleton organization (green), molecular transport (pink), and metabolism (cyan) are significantly altered in 1-year old R4344Q myocardia (*n* = 3) compared to isogenic gender- and age-matched control animals (*n* = 3). The *p*-value for each process is represented by the height of the respective bar, after being transformed into the negative logarithmic value with the base of 2. The number of proteins whose expression levels are altered in each process are denoted in the relevant columns.

Cardiac function and disease development, electropotential regulation, cytoskeletal organization, molecular transport, and metabolism were the main cellular processes that were significantly affected in the R4344Q female myocardium compared to wild-type (Figure 1). Among the 181 proteins whose expression levels were significantly altered in these cellular processes, some are well known to regulate cardiac homeostasis while others are currently understudied (Tables 1–6). Herein, we discuss the identified altered cellular processes and key relevant proteins aiming to provide a mechanistic interpretation of the arrhythmic phenotype developed by the R4344Q female animals in response to the physiological and irreversible process of aging. We followed three rules in the selection of proteins or families of proteins to discuss: (1) they are involved in several processes and/or sub-processes, (2) alterations in their expression levels or post-translational modifications have been linked to cardiac pathology, and (3) they directly or indirectly associate with obscurin. Of note, proteins that are involved in many processes and/or sub-processes are discussed under the ones for which more information regarding their role(s) is known.

**TABLE 1:** Altered expression levels of proteins involved in cardiac function and disease.

**Gene**	**Protein**	**% Change (95% Confidence interval)**	***P*-value**	**Unique peptides**	**Total peptides**	**Function or disease**
*CACNA1C*	Calcium channel, voltage-dependent, L-type, alpha 1C subunit	−16.4(−26.1,−5.6)	0.0151	9	9	Short QT syndrome; Long QT syndrome
*KCNQ1*	Potassium channel, voltage-gated, subfamily Q, member 1	−19.0(−31.6,−4.2)	0.0253	1	1	
*ANK1*	Small Ankyrin 1.5	44.0(2.0,103.3)	0.0426	1	3	Cardiac output
*DDAH1*	Dimethylarginine dimethylaminohydrolase 1	45.5(24.1,70.6)	0.0028	2	3	
*NPPA*	Natriuretic polypeptide type A	−37.4(−60.1,−1.8)	0.0447	3	3	
*PKG1*	Protein kinase, cGMP-dependent, type 1 (PKG1)	43.6(1.5,103.1)	0.0444	1	5	
*KCNQ1*	Potassium channel, voltage-gated, subfamily Q, member 1	−19.0(−31.6,−4.2)	0.0253	1	1	Familial atrial fibrillation
*NPPA*	Natriuretic polypeptide type A	−37.4(−60.1,−1.8)	0.0447	3	3	
*FHL2*	Four and a half LIM domains 2	−56.0(−79.7,−4.6)	0.0422	5	5	Relaxation of aortic ring tissue
*NPPA*	Natriuretic polypeptide type A	−37.4(−60.1,−1.8)	0.0447	3	3	
*CACNA1C*	Calcium channel, voltage-dependent, L-type, alpha 1C subunit	−16.4(−26.1,−5.6)	0.0151	9	9	Supraventricular tachycardia
*NPPA*	Natriuretic polypeptide type A	−37.4(−60.1,−1.8)	0.0447	3	3	
*GUCY1A2*	Guanylate cyclase 1, alpha 2	−16.2(−28.1,−2.5)	0.0317	1	1	Acute heart failure
*MME*	Membrane metallo endopeptidase	17.3(1.1,36.0)	0.0407	2	2	
*DYSF*	Dysferlin	−34.2(−49.5,−14.2)	0.0118	40	42	Early onset cardiomyopathy
*NPPA*	Natriuretic polypeptide type A	−37.4(−60.1,−1.8)	0.0447	3	3	Mild congestive heart failure

**TABLE 2:** Altered expression levels of proteins involved in electropotential regulation.

**Gene**	**Protein**	**% Change (95% Confidence interval)**	***P*-value**	**Unique peptides**	**Total peptides**	**Function**
*KCNJ11*	Potassium inwardly rectifying channel, subfamily J, member 11 (Kir6.2)	17.9 (0.9, 37.8)	0.0427	4	4	Transmembrane potential of cellular membrane
*KCNQ1*	Potassium channel, voltage-gated subfamily Q, member 1	−19.0 (−31.6, −4.2)	0.0253	1	1	
*PHB*	Prohibitin	19.8 (6.2, 35.2)	0.0142	15	15	
*PHB2*	Prohibitin 2	21.5 (3.4, 42.9)	0.0288	20	20	
*KCNQ1*	Potassium channel, voltage-gated subfamily Q, member 1	−19.0 (−31.6, −4.2)	0.0253	1	1	Flux of potassium
*NPPA*	Natriuretic polypeptide type A	−37.4 (−60.1, −1.8)	0.0447	3	3	
*NPPA*	Natriuretic polypeptide type A	−37.4 (−60.1, −1.8)	0.0447	3	3	Co-transport of chloride

**TABLE 3:** Altered expression levels of proteins involved in cytoskeleton organization, cellular assembly and maintenance.

**Gene**	**Protein**	**% Change (95% Confidence interval)**	***P*-value**	**Unique peptides**	**Total peptides**	**Function**
*CLIP1*	CAP-GLY domain containing linker protein 1	−16.3 (−29.3, −0.8)	0.0435	1	46	Stabilization of filaments
*EML2*	Echinoderm microtubule associated protein like 2	−28.9 (−40.7, −14.7)	0.0065	9	9	
*GALK2*	Galactokinase 2	−6.8 (−11.1, −2.4)	0.0138	2	2	
*GAP43*	Growth associated protein 43	−32.0 (−44.5, −16.6)	0.0063	4	4	
*KIFC3*	Kinesin family member C3	15.8 (1.4, 32.2)	0.0373	1	1	
*MAP1B*	Microtubule-associated protein 1B	−16.9 (−28.1, −3.9)	0.0242	9	9	
*ACTB*	Actin, beta	−11.7 (−22.0, −0.1)	0.0485	8	28	Microtubule dynamics
*CCL21A*	Chemokine (C-C motif) ligand 21A	−21.9 (−35.9, −4.7)	0.0260	1	1	
*CLIP1*	CAP-GLY domain containing linker protein 1	−16.3 (−29.3, −0.8)	0.0435	1	46	
*CRK*	v-crk avian sarcoma CT10 oncogene homolog	22.2 (4.4, 43.0)	0.0242	10	10	
*CTNND1*	Catenin, delta 1	−10.0 (−18.4, −0.8)	0.0397	1	13	
*CTSS*	Cathepsin S	−16.6 (−30.2, −0.4)	0.0470	1	1	
*DDAH1*	Dimethylarginine dimethylaminohydrolase 1	45.5 (24.1, 70.6)	0.0028	2	3	
*EIF4G2*	Eukaryotic translation initiation factor 4, gamma 2	24.2 (7.9, 43.1)	0.0130	14	14	
*EML2*	Echinoderm microtubule associated protein like 2	−28.9 (−40.7, −14.7)	0.0065	9	9	
*EPHA4*	Eph receptor A4	14.9 (2.1, 29.3)	0.0312	3	3	
*GALK2*	Galactokinase 2	−6.8 (−11.1, −2.4)	0.0138	2	2	
*GAP43*	Growth associated protein 43	−32.0 (−44.5, −16.6)	0.0063	4	4	
*GAPDH*	Glyceraldehyde-3-phosphate dehydrogenase	−11.8 (−21.3, −1.2)	0.0371	22	22	
*GIT1*	G protein-coupled receptor kinase-interactor 1	−10.2 (−17.3, −2.4)	0.0232	5	5	
*HDAC6*	Histone deacetylase 6	−14.0 (−24.5, −2.0)	0.0324	5	5	
*IDE*	Insulin degrading enzyme	6.4 (1.7, 11.4)	0.0188	8	8	
*KIF1C*	Kinesin family member 1C	52.6 (18.3, 96.8)	0.0100	5	6	
*KIFC3*	Kinesin family member C3	15.8 (1.4, 32.2)	0.0373	1	1	
*MAP1B*	Microtubule-associated protein 1B	−16.9 (−28.1, −3.9)	0.0242	9	9	
*MAPK8IP3*	Mitogen-activated protein kinase 8 interacting protein 3	22.5 (2.8, 46.0)	0.0325	7	12	
*MGLL*	Monoglyceride lipase	−14.6 (−27.0, −0.1)	0.0488	3	3	
*PKG1*	Protein kinase, cGMP-dependent, type 1 (PKG1)	43.6 (1.5, 103.1)	0.0444	1	5	
*PRPH*	Peripherin	−26.1 (−39.3, −10.1)	0.0129	9	13	
*PRUNE2*	Prune homolog 2	23.1 (6.9, 41.7)	0.0148	7	7	
*PTPN11*	Protein tyrosine phosphatase, non-receptor type 11	20.8 (9.1, 33.8)	0.0067	21	21	
*SIRPA*	Signal-regulatory protein alpha	17.8 (5.7, 31.3)	0.0138	2	2	
*SLK*	Ste20-like kinase	10.1 (2.8, 18.0)	0.0180	15	16	
*TUBB3*	Tubulin, beta 3	−31.4 (−49.5, −6.9)	0.0266	2	13	
*VLDLR*	Very low density lipoprotein receptor	7.6 (1.2, 14.3)	0.0291	8	8	
*DYSF*	Dysferlin	−34.2 (−49.5, −14.2)	0.0118	40	42	Damage of transverse tubule
*FHL2*	Four and a half LIM domains 2	−56.0 (−79.7, −4.6)	0.0422	5	5	Cell growth and differentiation; HCM and DCM

*FHL2 was not identified by the Mascot software as a cytoskeletal regulator, possibly due to its still understudied role. However, given that FHL2 is an integral component of the sarcomere, interacts with other major sarcomeric proteins (e.g., obscurin and titin), and has been implicated in the regulation of cardiomyocyte differentiation/growth and the development of hypertrophic and dilated cardiomyopathy (Liang et al., 2018), we included it as a regulator of cytoskeleton organization, cellular assembly and maintenance.*

**TABLE 4:** Altered expression levels of proteins involved in molecular transport.

**Gene**	**Protein**	**% Change (95% Confidence interval)**	***P*-value**	**Unique peptides**	**Total peptides**	**Function**
*ABAT*	4-aminobutyrate aminotransferase	−29.9 (−38.6, −19.9)	0.0018	8	8	Secretion of molecules
*CP*	Ceruloplasmin	40.0 (8.4, 80.9)	0.0217	1	22	
*KCNJ11*	Potassium inwardly rectifying channel, subfamily J, member 11 (Kir6.2)	17.9 (0.9, 37.8)	0.0427	4	4	
*KCNQ1*	Potassium channel, voltage-gated, subfamily Q, member 1	−19.0 (−31.6, −4.2)	0.0253	1	1	
*MGLL*	Monoglyceride lipase	−14.6 (−27.0, −0.1)	0.0488	3	3	
*NPPA*	Natriuretic polypeptide type A	−37.4 (−60.1, −1.8)	0.0447	3	3	
*PHB*	Prohibitin	19.8 (6.2, 35.2)	0.0142	15	15	
*PLIN2*	Perilipin2	16.5 (2.7, 32.2)	0.0280	5	5	
*POFUT2*	Protein *O*-fucosyltransferase 2	21.9 (1.3, 46.7)	0.0411	3	3	
*PKG1*	Protein kinase, cGMP-dependent, type 1 (PKG1)	43.6 (1.5, 103.1)	0.0444	1	5	
*PTPN11*	Protein tyrosine phosphatase, non-receptor type 11	20.8 (9.1, 33.8)	0.0067	21	21	
*RAB3B*	Rab3b	−30.3 (−46.0, −10.0)	0.0172	1	2	
*SIRPA*	Signal-regulatory protein alpha	17.8 (5.7, 31.3)	0.0138	2	2	
*TRAF3*	TNF receptor-associated factor 3	9.1 (0.3, 18.7)	0.0451	1	1	
*GOT2*	Glutamatic-oxaloacetic transaminase 2, mitochondrial	−16.8 (−30.0, −1.2)	0.0408	29	29	Transport of long chain fatty acids
*PLIN2*	Perillipin2	16.5 (2.7, 32.2)	0.0280	5	5	
*SLC27A4*	Solute carrier family 27	19.4 (0.9, 41.2)	0.0428	3	4	
*NPPA*	Natriuretic peptide type A	−37.4 (−60.1, −1.8)	0.0447	3	3	Flux of water

**TABLE 5:** Altered expression levels of proteins involved in metabolism.

**Gene**	**Protein**	**% Change (95% Confidence interval)**	***P*-value**	**Unique peptides**	**Total peptides**	**Function**
*ABAT*	4-aminobutyrate aminotransferase	−29.9(−38.6,−19.9)	0.0018	8	8	Catabolism of lipids
*ACOT7*	Acyl-CoA thioesterase 7	22.0(1.2,47.2)	0.0418	5	5	
*DDHD2*	DDHD domain containing 2	30.4(0.3,69.5)	0.0486	1	1	
*HEXB*	Hexosaminidase B	11.5(3.9,19.7)	0.0129	3	3	
*MGLL*	Monoglyceride lipase	−14.6(−27.0,−0.1)	0.0488	3	3	
*SLC27A4*	Solute carrier family 27	19.4(0.9,41.2)	0.0428	3	4	
*VLDLR*	Very low density lipoprotein receptor	7.6(1.2,14.3)	0.0291	8	8	
*ABAT*	4-aminobutyrate aminotransferase	−29.9(−38.6,−19.9)	0.0018	8	8	Metabolism of amino acids
*BCKDHB*	Branched chain ketoacid dehydrogenase E1, beta polypeptide	−18.6(−31.3,−3.5)	0.0284	5	5	
*DDAH1*	Dimethylarginine dimethylaminohydrolase 1	45.5(24.1,70.6)	0.0028	2	3	
*DDO*	D-aspartate oxidase	−55.4(−74.7,−21.4)	0.0167	2	2	
*GOT2*	Glutamatic-oxaloacetic transaminase 2, mitochondrial	−16.8(−30.0,−1.2)	0.0408	29	29	
*IVD*	Isovaleryl coenzyme A dehydrogenase	−12.5(−20.2,−4.1)	0.0156	19	19	
*MCCC2*	Methylcrotonoyl-coenzyme A carboxylase 2	−12.2(−20.3,−3.4)	0.0198	22	22	
*NPPA*	Natriuretic polypeptide type A	−37.4(−60.1,−1.8)	0.0447	3	3	
*PTS*	6-pyruvoyl-tetrahydropterin synthase	−13.6(−24.3,−1.4)	0.0375	2	2	
*ACOT7*	Acyl-CoA thioesterase 7	22.0(1.2,47.2)	0.0418	5	5	Metabolism of coenzyme A
*MCCC2*	Methylcrotonoyl-coenzyme A carboxylase 2	−12.2(−20.3,−3.4)	0.0198	22	22	
*SLC27A4*	Solute carrier family 27	19.4(0.9,41.2)	0.0428	3	4	
*HEXB*	Hexosaminidase B	11.6(3.9,19.7)	0.0129	3	3	Metabolism of N-acetylglucosamine
*NAGK*	*N*-acetylglucosamine kinase	−14.7(−21.0,−7.8)	0.0047	5	5	
*CST3*	Cystatin C	−32.6(−50.2,−8.7)	0.0225	3	3	Hydrolysis of protein fragments
*CTSS*	Cathepsin S	−16.6(−30.2,−0.4)	0.0470	1	1	
*CTSZ*	Cathepsin Z	50.0(15.8,94.1)	0.0121	2	2	
*FBXO6*	F-box protein 6	−18.8(−27.6,−8.8)	0.0075	2	2	
*HDAC6*	Histone deacetylase 6	−14.0(−24.5,−2.0)	0.0324	5	5	
*IDE*	Insulin degrading enzyme	6.4(1.7,11.4)	0.0188	8	8	
*MME*	Membrane metallo endopeptidase	17.3(1.1.36.0)	0.0407	2	2	
*TRAF3*	TNF receptor-associated factor 3	9.1(0.3,18.7)	0.0451	1	1	
*UBE2N*	Ubiquitin-conjugating enzyme E2N	−11.8(−20.0,−2.7)	0.0236	7	7	
*SLC25A20*	Solute carrier family 25 (mitochondrial carnitine/acylcarnitine translocase), member 20	16.7(9.2,24.6)	0.0029	14	14	Canitine acyl-carnitine translocase deficiency
*MGLL*	Monoglyceride lipase	−14.6(−27.0,−0.1)	0.0488	3	3	Hydrolysis of glycerol
*OXCT1*	3-oxoacid CoA transferase 1	−15.1(−24.1,−5.1)	0.0152	23	23	Metabolism of ketone body
*GAPDH*	Glyceraldehyde-3-phosphate dehydrogenase	−11.8(−21.3,−1.2)	0.0371	22	22	Production of glyceraldehyde- 3-phosphate

**TABLE 6:** Altered expression levels of proteins involved in various molecular pathways.

**Gene**	**Protein**	**% Change (95% Confidence interval)**	***P*-value**	**Unique peptides**	**Total peptides**	**Molecular Pathways**
*CYB5A*	Cytochrome b5 type A (microsomal)	−39.6 (−62.2, −3.5)	0.0405	4	4	γ-linolenate biosynthesis II
*SLC27A4*	Solute carrier family 27 (fatty acid transporter), member 4	19.4 (0.9, 41.2)	0.0428	3	4	
*ACOT2*	Acyl-CoA thioesterase 2	20.4 (4.3, 39.1)	0.0231	8	15	Stearate biosynthesis I
*ACOT7*	Acyl-CoA thioesterase 7	22.0 (1.2, 47.2)	0.0418	5	5	
*SLC27A4*	Solute carrier family 27 (fatty acid transporter), member 4	19.4 (0.9, 41.2)	0.0428	3	4	
*ACOT2*	Acyl-CoA thioesterase 2	20.4 (4.3, 39.1)	0.0231	8	15	Acyl-CoA hydrolysis
*ACOT7*	Acyl-CoA thioesterase 7	22.0 (1.2, 47.2)	0.0418	5	5	
*NUDT12*	Nudix (nucleoside diphosphate linked moiety X)-type motif 12	66.5 (15.9, 139.1)	0.0174	4	4	NAD salvage pathway II
*NMNAT3*	Nicotinamide nucleotide adenylyltransferase 3	18.9 (7.1, 32.0)	0.0100	4	4	NAD salvage pathway II and III
*IVD*	Isovaleryl coenzyme A dehydrogenase	−12.5 (−20.2, −4.1)	0.0156	19	19	Leucine degradation I
*MCCC2*	Methylcrotonoyl-coenzyme A carboxylase 2	−12.2 (−20.3, −3.4)	0.0198	22	22	
*HSD17B10*	Hydroxysteroid (17-beta) dehydrogenase 10	−10.2 (−15.3, −4.8)	0.0070	14	14	Fatty Acid β-oxidation
*IVD*	Isovaleryl coenzyme A dehydrogenase	−12.5 (−20.2, −4.1)	0.0156	19	19	
*SLC27A4*	Solute carrier family 27 (fatty acid transporter), member 4	19.4 (0.9, 41.2)	0.0428	3	4	
*PTPN11*	Protein tyrosine phosphatase, non-receptor type 11	20.8 (9.1, 33.8)	0.0067	21	21	Role of JAK2 in hormone-like cytokine signaling
*SIRPA*	Signal-regulatory protein alpha	17.8 (5.7, 31.3)	0.0138	2	2	
*NR1D1*	Nuclear receptor subfamily 1, group D, member 1	42.0 (2.2, 97.3)	0.0414	2	2	Circadian rhythm signaling

### Cardiac Function and Disease

Consistent with the myopathic and arrhythmic phenotype that the 1-year old female R4344Q mice develop, the expression levels of several proteins involved in cardiac output and relaxation or the development of short and long QT syndrome, atrial fibrillation, supraventricular tachycardia, hypertrophy, and heart failure are significantly altered. These include ion channels, enzymes, secreted peptides, membrane and sarcomeric proteins (Table 1). Given that cardiac function and disease is broad and all-encompassing, we will discuss key identified proteins in the respective (sub)processes they are involved.

#### Ca^2+^-Handling Proteins

The expression levels of two Ca^2+^-handling proteins were significantly altered in the 1-year old female R4344Q left ventricles, the L-type voltage-dependent Ca^2+^ channel alpha 1C subunit (Cacna1C) and small ankyrin 1.5 (sAnk1.5) (Table 1). In particular, the expression levels of Cacna1C were decreased (∼16%), whereas the expression levels of sAnk1 were increased (44%) compared to wild-type (Table 1). Activation of the L-type voltage-dependent Ca^2+^ channel by membrane depolarization is essential to induce Ca^2+^ release from the sarcoplasmic reticulum (SR) membranes primarily via the ryanodine receptor (RyR) (Grunnet, 2010). Assuming that the open-probability of the Cacna1C L-type Ca^2+^ channel remains unaltered in the R4344Q myocardia, its reduced expression levels would potentially result in delayed Ca^2+^ release from the SR. Interestingly, we did not observe such an effect in isolated R4344Q cardiomyocytes (Hu et al., 2017), suggesting that the ∼16% decrease in the expression levels of Cacna1C was not sufficient to notably delay SR Ca^2+^ release or that other compensatory mechanisms alleviated such an effect.

Moreover, sAnk1.5 is a transmembrane protein residing in the SR with its COOH-terminus extending in the myoplasm where it binds to the extreme COOH-terminus of obscurin-A at two sites with distinct affinities (Bagnato et al., 2003; Kontrogianni-Konstantopoulos et al., 2003; Busby et al., 2010). Loss-of-function studies have postulated that sAnk1.5 is essential for the assembly and sarcomeric alignment of the SR through its interaction with obscurin-A (Kontrogianni-Konstantopoulos et al., 2006; Lange et al., 2009). Contrary to our proteomics analysis, we did not find the expression levels of sAnk1.5 statistically increased in LV from 1-year old R4344Q female mice compared to wild-type via immunoblotting, although we did observe a clear trend (Hu et al., 2017). This discrepancy may be due to the different methods used, i.e., the qualitative nature and low sensitivity of immunoblotting *vs.* the quantitative nature and high sensitivity of proteomics (Aebersold et al., 2013), and/or the commonly observed inter-individual variability among isogenic animals of the same genotype that may obscure the identification of statistical differences.

In addition to binding obscurin-A, sAnk1.5 interacts directly with the SarcoEndoplasmic Reticulum Ca^2+^ ATPase 1 (SERCA1) and its major regulator sarcolipin (SLN) (Desmond et al., 2015), which are preferentially expressed in skeletal muscles (Shaikh et al., 2016); importantly, SLN is also present in healthy atrial cells and SERCA1 is upregulated in atrial cells in pathological states (Shaikh et al., 2016). sAnk1 reduces the apparent Ca^2+^ affinity of SERCA1, albeit to a lesser extent compared to SLN (Desmond et al., 2015), and ablates the inhibitory effect of SLN to SERCA1 activity (Desmond et al., 2017). Whether sAnk1.5 may play a similar regulatory role in the myocardium, by directly binding to SERCA2 and/or its major regulator phospholamban (PLN) is currently unknown, yet intriguing. Our previous work demonstrated that wild-type obscurin binds to PLN modestly, but this interaction is markedly enhanced in the presence of the R4344Q mutation (Hu et al., 2017). We therefore proposed that mutant obscurin might sequester PLN leading to SERCA2 disinhibition (Hu et al., 2017). If sAnk1.5 is a regulator of SERCA2, too, its up-regulation may contribute to the increased SERCA2 activity (∼40%) that we observed in the R4344Q myocardium in two ways: (1) through direct binding to PLN and ablation of its inhibitory effect on SERCA2 (similar to mutant obscurin) or (2) by being a less potent inhibitor of SERCA2 activity, and competing PLN away due to its excess amounts in the R4344Q myocardium. Experiments in our lab are currently under way to test these possibilities.

### Electropotential Regulation

Our proteomics studies also revealed alterations in the expression levels of proteins involved in electropotential regulation. Specifically, the expression levels of two potassium (K^+^) channels, K^+^ channel voltage-gated subfamily Q member 1 (Kcnq1) and K^+^ inwardly rectifying channel subfamily J member 11 (Kir6.2) were significantly decreased (∼19%) and increased (∼18%), respectively (Table 2). In the healthy myocardium, Kcnq1 is responsible for K^+^ extrusion during the repolarization phase (Finlay et al., 2017). Loss-of-function mutations in *KCNQ1* have been associated with long QT syndrome, ventricular tachycardia, ventricular fibrillation, extrasystoles and atrial flutter (Table 1) (Tester and Ackerman, 2014), while gain-of-function mutations have been linked to short QT syndrome, cardiac arrest, and atrial arrhythmia including atrial flutter and atrial fibrillation (Table 1) (Lee et al., 2017). Conversely, the K_ATP_ channel Kir6.2 is predominantly closed, but opens in response to cardiac stress (e.g., hypoxia or ischemia) to permit K^+^ efflux (Foster and Coetzee, 2016). The opening of Kir6.2 due to stress has been regarded as cardioprotective, since it leads to shortening of action potentials and suppression of Ca^2+^ entry into cardiomyocytes resulting in inhibition of contractility and reduced energy consumption (Foster and Coetzee, 2016). Alterations in the expression levels or open probability of K^+^ channels have been linked to deregulation of the electrochemical gradient of cardiomyocytes resulting in abnormal or asynchronous beating (Finlay et al., 2017). Given that the expression of Kir6.2 is increased by ∼18% in the R4344Q LV, if its open probability remains unaltered, it may at least partially account for the development of tachycardia (Hu et al., 2017) due to the shortened duration of action potentials.

In addition to K^+^ channels, proteins interacting with ion channels may also contribute to electropotential regulation. Prohibitin and prohibitin 2, interacting with the large-conductance Ca^2+^-activated Big K^+^ (BK) channel in chicken cochleae (Sokolowski et al., 2011), are up-regulated by ∼20% in the R4344Q obscurin model (Table 2). Their expression profiles are interdependent, as alterations in the levels of one are followed by the other. They form a multimeric complex consisting of 12–16 pairs of heterodimers in the inner mitochondrial membrane where they are responsible for organizing the mitochondrial genome and maintaining mitochondrial morphology. In addition to mitochondria, prohibitin and prohibitin 2 localize in the nucleus and the plasma membrane, serving as a transcriptional activator (prohibitin), regulator of chromatin morphology (prohibitin 2), and signal transducers (both proteins), whereas not much is known about their plasma membrane localization (Osman et al., 2009). In cardiomyocytes, overexpression of prohibitin protected cardiomyocytes from apoptosis against oxidative stress, and ameliorated diabetic cardiomyopathy in a type-2 diabetic rat model (Dong et al., 2016). Consistent with its protective role, the expression levels of prohibitin were decreased in a rat cardiac hypertrophy model induced by isoproterenol (Chowdhury et al., 2013). Given that knockdown of prohibitin in epithelial cells resulted in inhibition of Complex I of the mitochondrial electron transport chain and increased production of reactive oxygen species (ROS) (Schleicher et al., 2008), it is plausible that upregulation of prohibitin (and possibly prohibitin 2) in the R4344Q obscurin model is an adaptive mechanism of the mutant myocardium promoting cardiomyocyte survival by improving cellular respiration and diminishing ROS production.

Natriuretic polypeptide type A (NppA) is involved in electropotential regulation (Table 2) in addition to other processes (Tables 1, 4, 5), and its levels are significantly decreased (∼37%) in the R4344Q LV compared to wild-type controls. It indirectly inhibits the Na^+^/K^+^/2Cl^–^ co-transport in cardiomyocytes by stimulating guanylyl cyclase (GC) and increasing cytoplasmic cGMP levels, therefore reducing cell volume (Clemo and Baumgarten, 1995). Moreover, NppA has anti-proliferative and anti-hypertrophic effects in the heart by inhibiting the Mitogen Activated Protein Kinase (MAPK) axis (Song et al., 2015). Secretion of NppA from cardiac muscle to plasma is regulated by the Kir6.2 ATP-sensitive K^+^ channel, as Kir6.2-deficient atrial myocytes release higher levels of NppA post-mechanical stretch than wild-type cells (Saegusa et al., 2005). In accord with the decreased Nppa levels (∼37%) in R4344Q LV, Kir6.2 expression is increased (∼18%; Table 2). Given that the expression NppA is affected in the R4344Q LV alongside with its direct involvement in several pathophysiological cellular processes, it is not surprising that our proteomics analysis found many of them altered.

### Cytoskeletal Organization, Cellular Assembly and Maintenance

As obscurin is a *bona fide* cytoskeletal protein, it is not surprising that the proteomics analysis indicated alterations in several proteins involved in cytoskeletal organization (Table 3). Proteins involved in filament stabilization and microtubule dynamics were the main two subgroups that were significantly altered. Given that the expression profile and pathophysiological role of the identified proteins involved in filament stabilization are currently understudied in the heart, we will discuss select proteins involved in microtubule dynamics.

The expression levels of 23 proteins regulating microtubule dynamics were altered. As such, the levels of G protein-coupled receptor kinase-interactor 1 (Git1) and catenin-delta1 (Ichii and Takeichi, 2007; Cernohorska et al., 2016) that promote microtubule assembly were decreased ∼10% (Table 3). Conversely, the levels of Prune homolog 2 (Prune2), an interacting partner of Microtubule Associated Protein 6 (MAP6), shown to suppress microtubule stability in response to cold conferred by MAP6 (Arama et al., 2012), are increased by ∼23% (Table 3). While down-regulation of Git1 and catenin-delta1 and up-regulation of Prune2 suggest that the R4344Q myocardium favors microtubule disassembly, alterations in the expression levels of enzymes involved in the post-translational regulation of microtubules suggest a contrary scenario. Specifically, the levels of Histone deacetylase 6 (HDAC6) are reduced by ∼14%, whereas the levels of Protein tyrosine phosphatase non-receptor type 11 (Ptpn11) are increased by ∼21% (Table 3). Since HDAC6-mediated deacetylation of α-tubulin promotes microtubule destabilization (Hubbert et al., 2002) and gain-of-function of Ptpn11 reduces HDAC6-mediated microtubule deacetylation (Tien and Chang, 2014), the coincident down-regulation of HDAC6 and up-regulation of Ptpn11 in the R4344Q myocardium likely results in increased acetylation of α-tubulin and thus microtubule stabilization. In view of the pivotal role of microtubules in mechanotransduction in the healthy heart and their potential as a novel therapeutic target for the “stiff” heart (Chaldakov, 2018), characterization of microtubule structure and dynamics in the R4344Q myocardium is of high pathophysiological relevance, which our lab plans to investigate.

Tethered at the Z/I junction through its interaction with titin, Four and a Half Lim domains protein 2 (FHL2) interacts with cytoskeletal (including obscurin) and signaling proteins as well as metabolic enzymes (Liang et al., 2018). FHL2 has been postulated to suppress cardiac hypertrophy by inhibiting the MAPK/ERK and calcineurin/nuclear factor of activated T-cells (NFAT) signaling cascades (Liang et al., 2018). As such, mutations in FHL2 are associated with the development of HCM and DCM (Friedrich et al., 2014). In human patients with HCM, the mRNA and protein levels of FHL2 are reduced by >50% (Friedrich et al., 2014). In agreement with this, FHL2 is down-regulated by ∼56% (Table 3) in the R4344Q LV. Although the R4344Q myocardia do not undergo obvious hypertrophy under sedentary conditions by 1-year of age, they are arrhythmic, and develop hypertrophy at 3-months of age due to mild stress and dilation following sustained mechanical stress (Hu et al., 2017). It is therefore possible that the reduced levels of FHL2 contribute to these phenotypic manifestations although it is currently unknown whether up-regulation of the MAPK/ERK pathway takes place in the R4344Q hearts.

Moreover, dysferlin levels were notably reduced by ∼34% in the obscurin R4344Q myocardium (Table 3). Given that dysferlin is essential in maintaining and/or repairing sarcolemmal and transverse tubule integrity in striated muscle cells (McElhanon and Bhattacharya, 2018), and dysferlin-null mice develop early onset cardiomyopathy when subjected to stress exercise, it is possible that cardiomyocytes containing mutant obscurin are more susceptible to mechanical stress and pathological remodeling of the plasma membrane and internal membrane systems.

### Molecular Transport

The expression levels of several proteins involved in molecular transport are also altered in the obscurin R4344Q LV (Table 4). In addition to K^+^ channels and Nppa discussed above, proteins involved in iron and fatty acid transport and secretion of extracellular proteases may impact cellular metabolism and homeostasis. Ceruloplasmin is essential for converting ferrous (Fe^2+^) to ferric (Fe^3+^) to promote its transport and compartmentalization in the body. Up-regulation of ceruloplasmin by ∼40% (Table 4) may thus enhance iron transport in the obscurin R4344Q mice. Additionally, ceruloplasmin may carry out other physiological functions, such as scavenging of free radicals and copper transport (Wang and Wang, 2018). It was previously shown that up-regulation of ceruloplasmin may confer cardiac protection to isolated rat hearts subjected to ischemia and reperfusion (Atanasiu et al., 1995). Consistent with this, the expression levels of ceruloplasmin are up-regulated in heart failure patients, potentially as a compensatory response (Cabassi et al., 2014). Thus, up-regulation of ceruloplasmin may be an adaptive mechanism developed by the R4344Q model to ameliorate the cardiomyopathic pathology.

The expression levels of three proteins involved in the transport of long chain fatty acids are altered, too, including glutamic-oxaloacetic transaminase 2 (Got2), perilipin 2 (Plin2), and solute carrier family 27 (Table 4). Got2 and Plin2 upregulation in the heart has been shown to lead to accumulation of lipid droplets (Holloway et al., 2011; Ueno et al., 2017). Interestingly, the expression levels of solute carrier family 27 have been suggested to positively correlate with fatty acid uptake (Milger et al., 2006), but also lipid oxidation (Jeppesen et al., 2012). In the R4344Q myocardium, Got2 expression was down-regulated by 16.8%, whereas Plin2 and solute carrier family 27 expression was up-regulated by 16.5 and 19.4%, respectively (Table 4). Given that no prominent lipid droplet formation was observed in the hearts of these animals (our unpublished observations), it is likely that the altered expression levels of these proteins are not sufficient to lead to detectable lipid droplet accumulation by 1-year of age. Follow-up studies with older animals will assess the possibility of lipid accumulation as a function of aging in the R4344Q model.

In addition to the proteins discussed above, others involved in molecular transport have been shown to confer cardiac protection or exacerbate cardiac pathology. Among those, cardiac-specific overexpression of signal-regulatory protein alpha (Sirpα) has been suggested to attenuate pressure overload-induced cardiac hypertrophy in mice (Jiang et al., 2014), whereas mice treated with a pharmacological inhibitor of monoglyceride lipase (Mgll) exhibit increased infarct size and worsened cardiac function post-myocardial infarction (Schloss et al., 2019). Interestingly, while Sirpα is up-regulated by 17.8%, Mgll is down-regulated by 14.6% in the R4344Q myocardium (Table 4). Moreover, mice with cardiac-specific deletion of TNF receptor-associated factor 3 (TRAF3) showed significantly reduced cardiac hypertrophy and dysfunction 4-weeks after aortic banding, whereas transgenic mice overexpressing TRAF3 developed severe cardiac hypertrophy in response to pressure overload (Jiang et al., 2015); notably, TRAF3 is modestly up-regulated (9.1%) in the R4344Q myocardia. Thus, the absence of apparent cardiac remodeling, yet the development of arrhythmia in 1-year old R4344Q animals (Hu et al., 2017), appears to be modulated in a complex manner by the concomitantly altered expression of several positive and negative regulators of cardiac (mal)adaptation.

### Metabolism

Proteins involved in the regulation of metabolic pathways are affected in the R4344Q myocardia, too (Table 5). With the exception of 4-aminobutyrate aminotransferase (Abat) and Mgll whose expression levels are down-regulated, the remaining proteins involved in lipid catabolism are up-regulated (Table 5). Mgll contributes to the catabolism of triglycerides and monoglycerides, and has been implicated in suppressing the inflammatory response post-myocardial infarction (Schloss et al., 2019). Consistent with this, pharmacological inhibition of Mgll exacerbates cardiac pathology post myocardial infarction, as discussed above (Schloss et al., 2019). In contrast to a general up-regulation of proteins involved in lipid catabolism, proteins involved in amino acid metabolism are down-regulated, with the exception of dimethylarginine dimethylaminohydrolase 1 (Ddah1), which was significantly up-regulated (45.5%; Table 5). Ddah1 degrades asymmetric dimethylarginine (ADMA), which inhibits nitric oxide synthase (NOS) and may serve as a marker of cardiovascular disease (Aldamiz-Echevarria and Andrade, 2012). In response to myocardial ischemia, Ddah1 is up-regulated in a canine model, along with reduced ADMA levels in the myocardial interstitial fluid (Zhang et al., 2017). Mice with cardiac-specific deletion of Ddah1 exhibited exacerbated LV hypertrophy, increased ventricular fibrosis, and reduced ejection fraction and fractional shortening in response to transverse aortic constriction and acute myocardial infarction (Xu et al., 2017; Hou et al., 2018). Moreover, Ddah1-null cardiomyocytes showed increased apoptosis when subjected to oxidative stress (Hou et al., 2018). Consistent with this, over-expression of Ddah1 in heart transplants of recipient mice led to attenuation of oxidative stress and suppression of graft coronary artery disease (Tanaka et al., 2005). Thus, up-regulation of Ddah1 may be a compensatory mechanism preventing cardiomyocyte hypertrophy and/or apoptosis in the R4344Q model.

**FIGURE 2:**
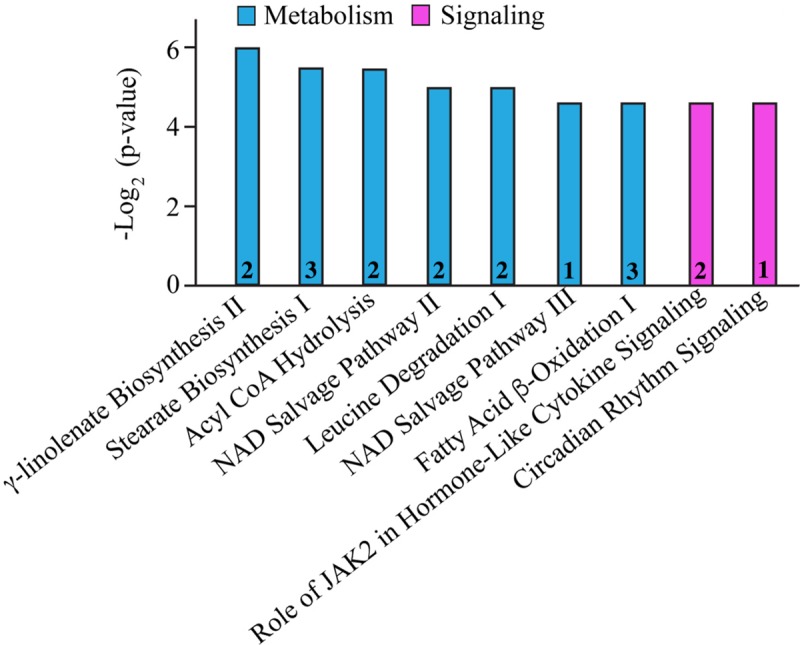
Molecular pathway annotation of proteins with altered expression levels in left ventricles of 1-year-old knock-in female animals. The expression levels of proteins involved in cellular metabolism (cyan) and signaling (pink) pathways are significantly altered in 1-year-old R4344Q myocardia (*n* = 3) compared to isogenic gender- and age-matched control animals (*n* = 3). The *p*-value for each molecular pathway is represented by the height of the respective bar after being transformed into the negative logarithmic value with the base of 2. The number of proteins whose expression levels are altered in each pathway are denoted in the relevant columns.

In addition to the alterations in major cellular processes discussed above, we also identified affected molecular pathways. As such, biosynthesis and/or degradation pathways of fatty acids and amino acids, cytokine signaling, circadian rhythm, and the NAD salvage pathway were identified (Figure 2 and Table 6). Notably, with the exception of Cytochrome b5 type A (Cyb5a), Nudix (nucleoside diphosphate linked moiety X)-type motif 12 (Nudt12), Nicotinamide nucleotide adenylyltransferase (Nmnat3) and Nuclear receptor subfamily 1 group D (Nr1d1), all other proteins participating in these pathways contribute to additional cellular processes, discussed in Tables 1–5. Although minimal information is available regarding the function of Cyb5a, Nudt12, and Nr1d1 in cardiac pathophysiology, Nmnat3 was previously suggested to confer anti-hypertrophic effects in response to angiotensin II induced cardiac hypertrophy (Yue et al., 2016). Thus, up-regulation of Nmnat3 (∼19%) may protect the R4344Q knock-in mice from developing hypertrophy under sedentary conditions.

### Altered Phosphorylation Levels

Moreover, we also examined whether the R4344Q LV exhibited alterations in protein post-translational modifications. Although several modifications were tested (e.g., glycosylation and glutathionylation), alterations in phosphorylation were the only significant ones. Two protein kinases (protein kinase cGMP-dependent type 1, PKG1, and Ste20-like kinase, Slk) and one protein phosphatase (protein tyrosine phosphatase, non-receptor type 11, Ptpn11) exhibited increased expression levels in the R4344Q myocardium (Tables 1, 3, 4, 6). PKG1 phosphorylates multiple substrates in the heart including cardiac myosin binding protein-C (cMyBP-C; Thoonen et al., 2015), transient receptor potential canonical channel 6 (TRPC6; Koitabashi et al., 2010), regulator of G-protein signaling subtype 4 (RGS4; Tokudome et al., 2008), and the BK channel, enhancing its activity (Frankenreiter et al., 2017). Although no direct phosphorylation on the L-type voltage-gated Ca^2+^ channel, Cacna1C, by PKG1 has been reported, it has been suggested that a PKG1-mediated pathway may suppress its open probability (Schroder et al., 2003). Thus, PKG1 may modulate cardiac contractility and output via the coordinated regulation of the activity of multiple ion channels in the heart. Consistent with this, PKG1 appears to have a cardioprotective role in the heart by attenuating hypertrophy and decreasing the extent of fibrosis via inhibition of phosphodiesterase 5 (PDE5) (Nishida et al., 2010). Given the ∼40% upregulation of PKG1 in the R4344Q LV, we speculate that it is one of the compensatory responses of the mutant myocardium contributing to preserved morphology and accounting for the presence of mild fibrosis (Hu et al., 2017). Slk, the second kinase whose expression is altered in the R4344Q myocardium, regulates microtubule organization and focal adhesion turnover through phosphorylation of dynactin and paxillin, respectively (Zhapparova et al., 2013). Up-regulated by 10%, Slk may modulate cytoskeletal organization in the R4344Q myocardium, alongside with other proteins involved in microtubule dynamics that exhibit altered expression. Moreover, the only phosphatase that is altered in the presence of the R4344Q mutation, Ptpn11, is also up-regulated by 20.8%. In cardiac development, Ptpn11 is required for maintenance of cardiac progenitor cells, as inhibition of Ptpn11 prevents cardiac differentiation (Langdon et al., 2007). Overexpression of wild-type Ptpn11 in neonatal rat ventricular cardiomyocytes (NRVM) failed to increase cell length in response to leukemia inhibitory factor (LIF) known to cause hypertrophy, whereas overexpression of a dominant-negative form of Ptpn11 led to attenuated cell elongation in response to LIF (Nakaoka et al., 2010). Moreover, Ptpn11 constitutively dephosphorylates focal adhesion kinase (FAK) and prevents cardiac hypertrophy, as down-regulation of Ptpn11 in NRVM prevented association of Ptpn11 with FAK leading to hypertrophy (Marin et al., 2008). Thus, the ∼20% upregulation of Ptpn11 that we observed in the R4344Q myocardium may aid along with PKG1 in the prevention of a hypertrophic maladaptive response in the R4344Q myocardium under sedentary conditions.

**FIGURE 3:**
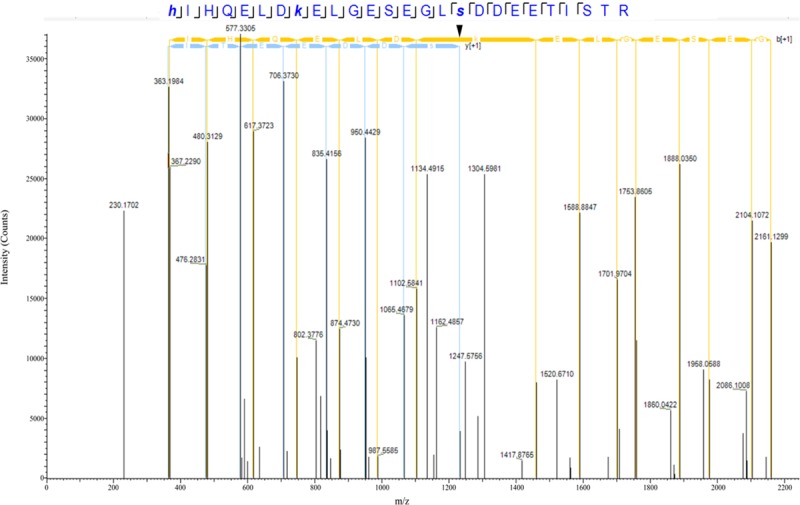
Identification of a novel phosphorylation site in sAnk1. The spectrum of the identified sAnk1 phosphorylated peptide is shown with the phospho-Ser55 peak denoted with an arrowhead.

Our proteomics screening identified alterations in the phosphorylation profile of sarcomeric and Ca^2+^ associated proteins, too (Table 7). As such, myosin heavy chain 6 (Myh6) showed increased phosphorylation levels (∼25%) on Ser1309 in the R4344Q LV compared to wild-type. Ser1309 is located in the coiled-coil region of the myosin tail and has been previously reported as a phosphosite in the murine heart downstream of the β-adrenergic receptor pathway; however, the responsible kinase and functional significance of this modification are still uncharacterized (Lundby et al., 2013). The Ca^2+^ associated proteins sAnk1.5 and junctophilin 2 (Jph2) exhibited alterations in their phosphorylation status, too. sAnk1.5 residing in the SR membranes displayed a ∼38% increase in the phosphorylation levels of Ser55 (NP_001297366.1; Figure 3). To our knowledge, this is the first study reporting regulation of sAnk1.5 via phosphorylation. Although the impact of Ser55 phosphorylation is currently elusive, it is possible that it may regulate the dynamic binding of sAnk1.5 to obscurin, SERCA and/or SLN/PLN (Bagnato et al., 2003; Kontrogianni-Konstantopoulos et al., 2003; Desmond et al., 2017) (please see above). Contrary to sAnk1.5, Jph2 that links the SR with the transverse tubules (Beavers et al., 2014) exhibited reduced phosphorylation (38%) at Ser597. Ser597 has been previously reported as a phosphosite of Jph2 (Lundby et al., 2013) although neither the responsible kinase nor the biological importance of this modification has been identified. Recently, striated preferentially expressed gene (SPEG) kinase, a homolog of the obscurin kinase isoform, was found to directly interact with Jph2 (Quick et al., 2017). Conditional loss of SPEG in murine hearts resulted in reduced overall Ser phosphorylation levels of Jph-2, but the affected phosphosites are not known (Quick et al., 2017). Lastly, the phosphorylation levels of Kelch domain containing 7A (Klhdc7a) and ubiquitin-associated protein 2-like (Ubap2l) proteins were drastically altered in R4344Q LV with Klhdc7a exhibiting a ∼195% increase in the phosphorylation levels of Ser361 and Ubap2l displaying a ∼53% decrease in the phosphorylation levels of Ser487 compared to wild-type; of note, both of these phospho-sites have been previously reported in the literature (Klhdc7a^1^ and Ubap21^2^). Currently, no study has addressed the role(s) of Klhdc7a. On the other hand, there are several reports on Ubap2l implicating it in cancer progression, and specifically in cell proliferation and epithelial to mesenchymal transition. Although the role of Ubap2l in the mammalian heart remains to be elucidated, it is reasonable to postulate that it might be involved in growth and/or hypertrophic responses. Given the up-regulation of Pkrg1 and Slk kinases, it is tempting to speculate that they may directly or indirectly mediate at least some of these phosphorylation events, a hypothesis that we plan to address.

**TABLE 7:** Altered phosphorylation levels of identified proteins.

**Gene**	**Protein**	**Site**	**Domain**	**% Change (95% Confidence interval)**	***P*-value**
*ANK1*	Small ankyrin 1.5	Ser55	Non-modular	38.3 (4.7, 82.8)	0.0319
*KLHDC7A*	Kelch domain containing 7A	Ser361	Non-modular	194.1 (70.2, 408.2)	0.0054
*MYH6*	Myosin, heavy polypeptide 6, cardiac muscle, alpha	Ser1309	Light meromyosin (LMM)	24.9 (3.6, 50.5)	0.0299
*UBAP2l*	Ubiquitin-associated protein 2-like	Ser487	Non-modular	−53.4 (−72.3, −21.6)	0.0152
*JPH2*	Junctophilin 2	Ser597	Non-modular	−38.4 (−61.1, −2.5)	0.0428

Taken together, our detailed proteomics study revealed alterations in the expression levels of major proteins involved in cardiac homeostasis, Ca^2+^ handling, cytoskeletal organization, ion and molecular transport, and metabolism. Given the presence of a single amino acid substitution (R4344Q) in a protein that may contain 6,600–8,000 amino acids depending on the isoform, it is simultaneously striking and perplexing to consider the identified molecular (this study) and functional (Hu et al., 2017) consequences. Although obscurin has been implicated in several cellular processes (Wang et al., 2018), its unequivocal contribution to the organization and cytoskeletal anchoring of the SR membranes and Ca^2+^ regulation (Bagnato et al., 2003; Kontrogianni-Konstantopoulos et al., 2003; Lange et al., 2009; Hu et al., 2017; Randazzo et al., 2017) strongly suggest that alterations in the expression and phosphorylation levels of Ca^2+^ handling proteins, ion channels and relevant enzymes may underlie the arrhythmic phenotype observed as a function of aging and the cardiac remodeling in response to stress (Hu et al., 2017). However, given the identified alterations in proteins involved in additional cellular processes for which there is some (e.g., microtubule dynamics; (Randazzo et al., 2013) or no (e.g., metabolism) evidence for the involvement of obscurin, it is appealing to hypothesize that obscurin may have new (previously unidentified) roles in the myocardium. Further work will assess this intriguing possibility, especially given the escalating number of *OBSCN* missense mutations associated with different forms of congenital cardiomyopathy (Grogan and Kontrogianni-Konstantopoulos, 2018).

Although our comprehensive proteomics analysis yielded a number of important and novel findings, we are cognizant of potential limitations of our study, including: (1) the relatively small number of animals that was analyzed per genotype, (2) the lack of relevant human biopsies, and (3) the examination of homozygous, rather than heterozygous, R4344Q animals, given that the mutation exhibits a dominant inheritance in humans.

Variability in isogenic animals is indeed a major concern for “omics” studies, but also a reality, whether a small or a large cohort is used. Although the source of variability is not well-understood, studies suggest that it might originate from random genetic variations despite extensive inbreeding, environmental influences such as intrauterine position of embryos or feeding hierarchy and maternal care of newborns as well as stochastic events affecting molecular and cellular processes (Loos et al., 2015). As such, there has been a recent tendency to perform individualized sample analysis, especially when human biopsies are used, refraining from collating and averaging data, a tactic that has likely obscured important idiosyncratic alterations. At this time, we are not aware of available cardiac biopsies that carry the obscurin R4344Q mutation or the disease progression and life-span of the affected patients, therefore evaluation of our preclinical mouse model appears to be the most optimal approach.

Moreover, our earlier (Hu et al., 2017) and current studies have focused on the homozygous population of the R4344Q model although the monoallelic presence of the mutation in humans is linked to HCM. Examination of the heterozygous R4344Q population under sedentary conditions did not reveal any cardiac pathology. This is not an uncommon phenomenon, where disease severity manifests to different extents between humans and the respective preclinical models, likely due to the phylogenetic distance of humans and rodents, species-specific differences, and the presence of non-controlled environmental influences and stresses that humans are subjected to (Camacho et al., 2016).

Notwithstanding the aforementioned limitations, our comprehensive proteomics analysis provides important insights about the molecular and cellular alterations that take place in the R4344Q myocardium by confirming some obvious ones and unraveling some new and unexpected ones to be interrogated in the future.

## Data Availability Statement

The raw data supporting the conclusions of this article will be made available by the authors, without undue reservation, to any qualified researcher.

## Ethics Statement

The animal study was reviewed and approved by Institutional Animal Care and Use Committees of the University of Maryland School of Medicine.

## Author Contributions

AK-K conceived the study. L-YH and AK-K designed the study and wrote the manuscript. L-YH performed the experiments and analyzed the data.

## Conflict of Interest

The authors declare that the research was conducted in the absence of any commercial or financial relationships that could be construed as a potential conflict of interest.

## Footnotes

1 https://www.uniprot.org/uniprot/A2APT9

2 https://www.uniprot.org/uniprot/Q80x50

## Supplementary Material

FILE S1 Relative abundance of proteins identified in the R4344Q-obscurin myocardia compared to wild-type.
